# DNA Origami-Constructed Nanotapes for Sunitinib Adsorption and Inhibition of Renal Clear Carcinoma Cells

**DOI:** 10.1021/acsomega.4c03091

**Published:** 2024-07-29

**Authors:** Lin Li, Xuxiang Yao, Pengyao Wei, Dongdong He, Qiaojiao Ding, Bing Bai, Xiuyi Lv, Akinori Kuzuya, Yuling Wang, Kerong Wu, Kaizhe Wang, Jianping Zheng

**Affiliations:** † Ningbo Key Laboratory of Biomedical Imaging Probe Materials and Technology, Ningbo Cixi Institute of Biomedical Engineering, Ningbo Institute of Materials Technology and Engineering of Chinese Academy of Sciences, Ningbo 315300, P. R. China; ‡ University of Chinese Academy of Sciences, Beijing 100049, P. R. China; § The First Affiliated Hospital of Ningbo University, Ningbo University, Ningbo 315300, P. R. China; ∥ Faculty of Chemistry, Materials, and Bioengineering, Kansai University, 3-3-35 Yamate, Suita, Osaka 564-8680, Japan; ⊥ School of Natural Sciences, Faculty of Science and Engineering, Macquarie University, Sydney, NSW 2109, Australia

## Abstract

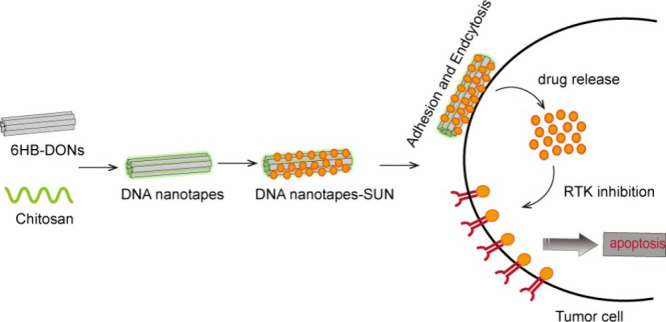

Sunitinib (SUN) is a first-line drug for the treatment of renal clear carcinoma cells by targeting receptor tyrosine kinases (RTK) on the cell membrane. However, the effective delivery of SUN to the cell membrane remains a significant challenge. In this study, we fabricated precisely structured DNA nanotapes with strong surface SUN adhesion, enabling RTK inhibition of renal clear carcinoma cells. In our design, the precisely assembled linear topological six-helical-bundle DNA origami serves as the framework, and positively charged chitosan is adsorbed onto the DNA origami surface, thereby forming DNA nanotapes. The SUN was efficiently loaded onto the surface of the DNA nanotapes by electrostatic interaction. We found that DNA nanotapes exhibit excellent stability in serum. Importantly, DNA nanotapes carrying SUN can achieve prolonged cell membrane retention and inhibit RTK, thereby enhancing cytotoxicity toward 786-0 cells. Taken together, this study provides a promising candidate platform for the efficient delivery of cell membrane receptor inhibitors in anticancer therapy.

## 1 Introduction

Renal carcinoma is the second most common malignancy of the urinary system, which is classified pathologically into clear cell carcinoma, papillary cell carcinoma, and chromophobe cell carcinoma.^1,2^ Among them, renal clear cell carcinoma accounts for approximately 70 to 75% of all renal malignancies.^3,4^ Therapeutic methods such as radiotherapy,^5^ chemotherapy,^6^ and immunotherapy^7^ show poor effects in patients with renal clear cell carcinoma. Targeted drugs show significant therapeutic benefits by blocking the signaling pathways of classical pathways.^8−10^ Among them, sunitinib is a multitarget RTK inhibitor that binds to RTKs on the cell membrane, inhibiting their tyrosine kinase activity.^11,12^ Sunitinib can inhibit tyrosine kinase activity and significantly inhibit tumor angiogenesis, which inhibits tumor growth. Interestingly, tyrosine kinase inhibitors have also been shown to directly inhibit the proliferation of tumor cells expressing tyrosine kinases such as VEGFR.^13−15^ In particular, the receptor tyrosine kinase is also expressed in renal cell carcinoma cells, and sunitinib can directly induce apoptosis in renal cell carcinoma cells.^16^ However, SUN is categorized in the lower bioavailability class IV using the biopharmaceutical classification system,^17^ owing to low permeability and water solubility. Meanwhile, SUN has also caused a variety of adverse reactions in clinical practice, including hypertension, diarrhea, vomiting, and hand–foot syndrome as well as white hair and, more seriously, damage to leucocytes and platelets due to its dispersive cell delivery.^18,19^ Therefore, it is critical to establish comprehensive strategies to improve the integrated delivery of SUN to tumor cells.

The unique physical and chemical properties of nanomaterials can enhance the efficiency of drug delivery. Thus far, numerous strategies proposed to deliver SUN molecules for enhancing bioavailability and delivery efficiency have been reported.^20,21^ For example, Francesca et al.^22^ used liposomes as delivery systems and then encapsulated SUN inside the liposomes, but it was difficult to achieve the accumulation of SUN on the cell membrane. Shreya et al.^23^ encapsulated SUN into the mesoporous channels of mesoporous silica nanoparticles and released them in response to pH stimulation. This strategy may enhance release from the extracellular environment, but it cannot achieve effective accumulation on the cells. Jayapal et al.^24^ used chitosan nanoparticles formed by cross-linking chitosan with sodium tripolyphosphate cross-linker to load SUN. However, the shape and size of nanoparticles formed by this method are uncontrollable. Taken together, the uncontrolled size of synthetic materials and the limited accumulation of SUN on cell membranes still hinder their wide application in clinics.

DNA origami as polyanion nanostructures, constructed from endogenous nucleic acid materials, with intrinsic biocompatibility, have been increasingly employed for developing novel drug delivery systems.^25−29^ More importantly, DNA nanostructures have demonstrated unprecedented precision in structural, surface topography control by exploiting Watson–Crick base-pairing self-assembly.^30,31^ So far, DNA nanostructure-based drug delivery systems have been proposed to deliver a range of drugs via loading on the DNA nanostructure surface. For example, based on the programmable three-dimensional DNA origami nanostructures, Zhong et al.^32^ screened three different DNA origami surfaces for loading Pt(IV) prodrugs to enhance the cellular delivery capacity. Joona et al.^33^ utilized virus capsid protein to encapsulate DNA origami through electrostatic interactions, achieving efficient delivery to cells. Based on a precisely addressable surface of DNA origami, Ge et al.^34^ modified multiple ligands on the surface of DNA origami to bind to tumor cell membrane surface receptors, thereby improving their target ability.

Compared with other DNA structures, six-helical-bundle DNA origami nanostructures (6HB-DONs) could offer good stability in physiological environments such as cell culture medium^35^ and prolonged circulation of the half-life in vivo.^36^ In this study, we constructed DNA nanotapes based on 6HB-DON nanostructures for SUN adsorption and delivery, achieving long retention and RTK inhibition on the cell membrane, thereby increasing drug efficacy on renal clear carcinoma cells. Briefly, DNA nanotapes were constructed using well-defined long linear topological 6HB-DONs as framework cores and chitosan as an adhesive, with the polyanion surface of the 6HB-DONs coated with the cationic polysaccharide chitosan. SUN was loaded onto the surface of DNA nanotapes through electrostatic interactions with chitosan. DNA nanotapes exhibited a high drug-loading capacity and enhanced cellular delivery efficiency. Importantly, the DNA nanotapes exhibited strong adhesion and binding to renal clear carcinoma cells, leading to an enhanced drug concentration on the cell membrane and improving antitumor activity. This study provided a promising platform for cell membrane receptor inhibitor delivery.

## 2 Materials and Methods

### 2.1 Materials and Reagents

A single-stranded DNA scaffold (M13 ssDNA) was purchased from Cnbioruler (Suzhou, China). All oligonucleotides were purchased from Sangon Biotech (Shanghai, China). Chitosan oligosaccharide lactate (Mn ≈ 4000–6000, >90% deacetylated, 60% composition oligosaccharide) and sunitinib (with a purity of more than 99 wt %) were obtained from Sigma-Aldrich (USA). Antibiotics (penicillin/streptomycin) and glutathione were purchased from Beyotime Biotechnology. 1640 medium, phosphate-buffered saline (PBS), and fetal bovine serum (FBS) were purchased from Gibco BRL (Grand Island, USA). Cell Counting Kit-8 was purchased from Glpbio (USA). All other chemicals or materials were purchased from Sigma-Aldrich and used as received unless stated otherwise. Ultrapure water was used in all experiments.

### 2.2 Synthesis of 6HB-DONs

6HB-DONs were designed and assembled according to Rothemund’s and Yan’s methods.^26,31^ Briefly, M13mp18 and 168 staple strands were assembled in 1× TAE/Mg^2+^ buffer (40 mM tris, 20 mM acetic acid, 2 mM EDTA, and 12.5 mM magnesium acetate, pH 8.3), with a M13mp18/stapleton ratio of 1:5. The mixed solution was annealed according to the following annealing program: 95 °C for 5 min, 85 to 65 °C for 1 h, and then slow cooling from 65 to 25 °C during a period of 8 h. The annealed solution was centrifuged using a 100 kDa MWCO ultrafiltration tube at a speed of 3000*g* for 15 min, and this process was repeated three times to remove excess unassembled staple strands. The purified DNA nanotube origami was measured by the absorbance peak at 260 nm using a UV spectrophotometer (Cary300, Agilent).

### 2.3 Synthesis of Nanotapes

To prepare DNA nanotapes at 0.05 N/P, 3.6 μL of chitosan (stock concentration of 2.5 μg/mL) was added to 6.4 μL of tris buffer. Then, an additional 10 μL of 6HB-DONs (containing 1 nmol of phosphate) was added.

### 2.4 Synthesis of Nanotapes-SUN

DNA nanotapes were mixed with SUN in a molar ratio of 1:1000 at 20 °C under a speed of 500 ramps for 3 h. The DNA nanotape-SUN was purified using a 100 kDa MWCO ultrafiltration tube at a speed of 3000*g* for 15 min, and this process was repeated three times to remove unloaded sunitinib. The free sunitinib in the supernatant was isolated and quantified by measuring the absorption of sunitinib at 430 nm by a fluorescence spectrophotometer, according to the standard curve. The sunitinib loading content and efficiency of loading into the DNA nanotapes were calculated as follows:
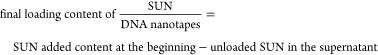

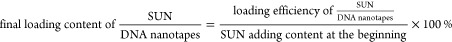


Each DNA sample (10 μL) was mixed with 6× loading buffer (2 μL) and analyzed by using 1.2% agarose gel electrophoresis at 110 mV for about 45 min in 1× TAE buffer. The bands were visualized by UV exposure and photographed by a gel imaging system (ChemiDoc, Biored).

### 2.5 AFM Imaging

A 5 μL sample solution was adsorbed on a freshly cleaved mica surface for 10 min. Subsequently, the mica surface was washed with ultrapure water and dried with a nitrogen gun. AFM imaging was performed using the ScanAsyst mode in Dimension FastScan AFM (Bruker).

### 2.6 Zeta Potential Analysis

The ζ-potential values of 6HB-DONs and DNA nanotapes were measured using dynamic light scattering (DLS) on a Litesizer 500 (Anton Paar Instruments). Samples with a 6HB-DONs concentration of 50 nM were measured three times at 25 °C. The data was analyzed using the built-in multimodal size distribution software of the instrument and expressed as the mean ± SD.

### 2.7 UV–Vis Spectroscopy Analysis

The absorbances of 6HB-DONs and DNA nanotape-SUN were measured by full wavelength scanning using a UV spectrophotometer (Cary300, Agilent).

### 2.8 Agarose Gel Electrophoresis Analysis

6HB-DONs and DNA nanotapes were separately incubated with 10% FBS in 1640 medium at 37 °C for 24, 48, and 72 h. Each sample (10 μL) was mixed with 6× loading buffer (2 μL) and analyzed using 1.2% agarose gel electrophoresis at 110 mV for about 45 min in 1× TAE buffer.

### 2.9 Cell Culture

The 786-0 cells were donated by the first affiliated hospital of Ningbo University. 786-0 cells were cultured with 10% FBS in 1640 medium under the conditions of 5% CO_2_ at 37 °C.

### 2.10 Confocal Imaging

786-0 cells (0.6 × 10^4^ in 200 μL of medium) were seeded in 35 mm confocal dishes and cultured overnight. Then cells were incubated with Cy5-labeled 6HB-DONs (Cy5, 500 nM; 6HB-DONs, 50 nM, 20 μL), Cy5-labeled DNA nanotapes (Cy5, 500 nM; 6HB-DONs, 50 nM, 20 μL), and Cy5-labeled DNA nanotape-SUN (Cy5, 500 nM; 6HB-DONs, 50 nM, 20 μL) in 200 μL of 1640 medium with 10% FBS for 3, 6, and 12 h at 37 °C. Next, the cells were incubated with Hoechst 33342 for 15 min. The cells were fixed with 4% paraformaldehyde for 15 min and then were washed with 1× PBS three times and imaged by laser confocal fluorescence microscopy (TCS SP8, Leica).

### 2.11 Flow Cytometry Analysis

786-0 cells (1.5 × 10^5^ in 1 mL of medium) were seeded in 35 mm dishes and cultured overnight. Then the cells were incubated with Cy5-labeled 6HB-DONs (Cy5, 500 nM; 6HB-DONs, 50 nM, 40 μL), Cy5-labeled DNA nanotapes (Cy5, 500 nM; 6HB-DONs, 50 nM, 40 μL), and Cy5-labeled DNA nanotape-SUN (Cy5, 500 nM; 6HB-DONs, 50 nM, 40 μL) in 400 μL with 10% FBS in 1640 medium for 12 h at 37 °C. The cells were centrifuged at 1000 rpm for 4 min and then resuspended in 1 mL of PBS for FACS analysis (LSRFortessa, Becton Dickinson). The data was processed with FlowJo software.

### 2.12 Cell Viability Assay

786-0 cells (1.5 × 10^5^ in 1 mL of medium) were seeded in a 96-well plate and cultured overnight. The cells were incubated with a mixing solution including 6HB-DONs, SUN, and DNA nanotape-SUN with 10% FBS in 1640 medium at 37 °C, separately. After 24 h of incubation, cells were washed with PBS and incubated with 50 μL of 1640 medium with 10% Cell Counting Kit-8 reagent for 2 h. Then the absorption was measured at 450 nm. 786-0 cells (1.5 × 10^5^ in 1 mL of medium) were seeded in a 96-well plate and cultured overnight. The cells were incubated with SUN (0, 10, 50, 100, 250, and 500 nM), DNA nanotape-SUN (concentration gradient of SUN 0, 10, 50, 100, 250, and 500 nM) containing 10% FBS at 37 °C. After 24 h of incubation, cells were washed with PBS and incubated with 50 μL of 1640 medium with 10% Cell Counting Kit-8 reagent for 2 h. Then the absorption was measured at 450 nm.

### 2.13 TUNEL Assay

786-0 cells (1.5 × 10^5^ in 1 mL of medium) were seeded in 35 mm dishes and cultured overnight. The cells were incubated with a mixing solution including 6HB-DONs, SUN, and DNA nanotape-SUN with 10% FBS in the 1640 medium at 37 °C, separately. After 24 h of incubation, cells were washed with PBS and were fixed with 4% paraformaldehyde for 15 min. The cells were incubated with 0.2% Triton X-100 for 10 min at 37 °C. Next, cells were equilibrated by incubation with 100 μL of TDT equilibration buffer for 30 min and then were incubated with 50 μL of labeling solution at 37 °C in the dark for 60 min. Finally, the cells were incubated with DAPI working solution at room temperature in the dark for 5 min and then were washed with 1× PBS three times and imaged by laser confocal fluorescence microscopy (TCS SP8, Leica).

### 2.14 Statistical Analysis

Statistical analysis was conducted using GraphPad Prism software. One-way ANOVA and the *t* test followed by multiple comparisons were used to determine the statistical differences between the groups. Quantitative data are presented as the mean ± SD, and **p < 0.01 and ***p < 0.001 were considered to be statistically significant.

## 3 Results and Discussion

### 3.1 DNA Nanotapes Design

**Figure 1:**
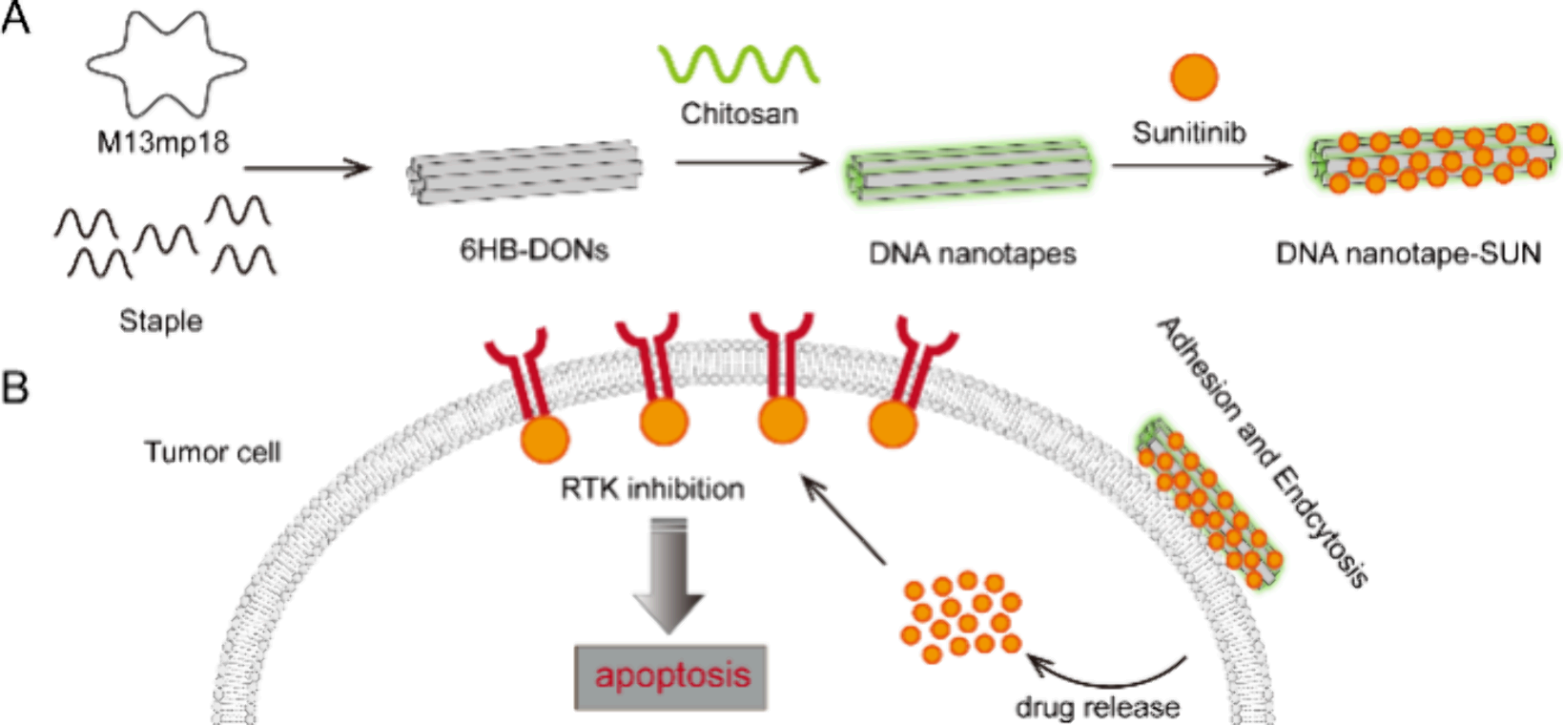
Schematic of DNA origami-based nanotapes for SUN adhesion and tumor cell inhibition. (A) Six-helical-bundle DNA origami nanostructure was constructed according to the DNA origami technique. Negatively charged DNA origami nanostructure interacting with positively charged chitosan forms DNA nanotapes through electrostatic interaction. DNA nanotapes were adsorbed with sunitinib to form DNA nanotape-SUN. (B) Schematic representation of utilizing the DNA nanotape-SUN for target cancer therapy.

The design principle for DNA nanotapes is illustrated in Figure 1. In this study, we utilized 6HB-DONs as the framework cores of DNA nanotapes, which possess a length of 380 nm and a linear structure. These 6HB-DONs were assembled from a 7249-nucleotide-long M13 bacteriophage genome DNA and multiple short single strands through a single-step annealing process.^37^ Additionally, chitosan, an FDA-approved biopolymer with high positive charge density and biocompatible, biodegradable, and nonimmunogenic properties, was incorporated into the DNA nanotapes. The chitosan was mixed with the 6HB-DONs and formed a layer of adhesive coating on the surface of the 6HB-DONs through electrostatic adsorption.^38^ Furthermore, the DNA nanotapes were able to efficiently load SUN through electrostatic interactions with chitosan on the surface.^24^ The elongated structure of DNA nanotapes enabled them to adhere to the negatively charged cell membrane, and the loaded SUN could be released for binding to receptor tyrosine kinases on the cell membrane, thereby achieving sustained cellular inhibition.

### 3.2 DNA Nanotape Synthesis and Characterization

**Figure 2:**
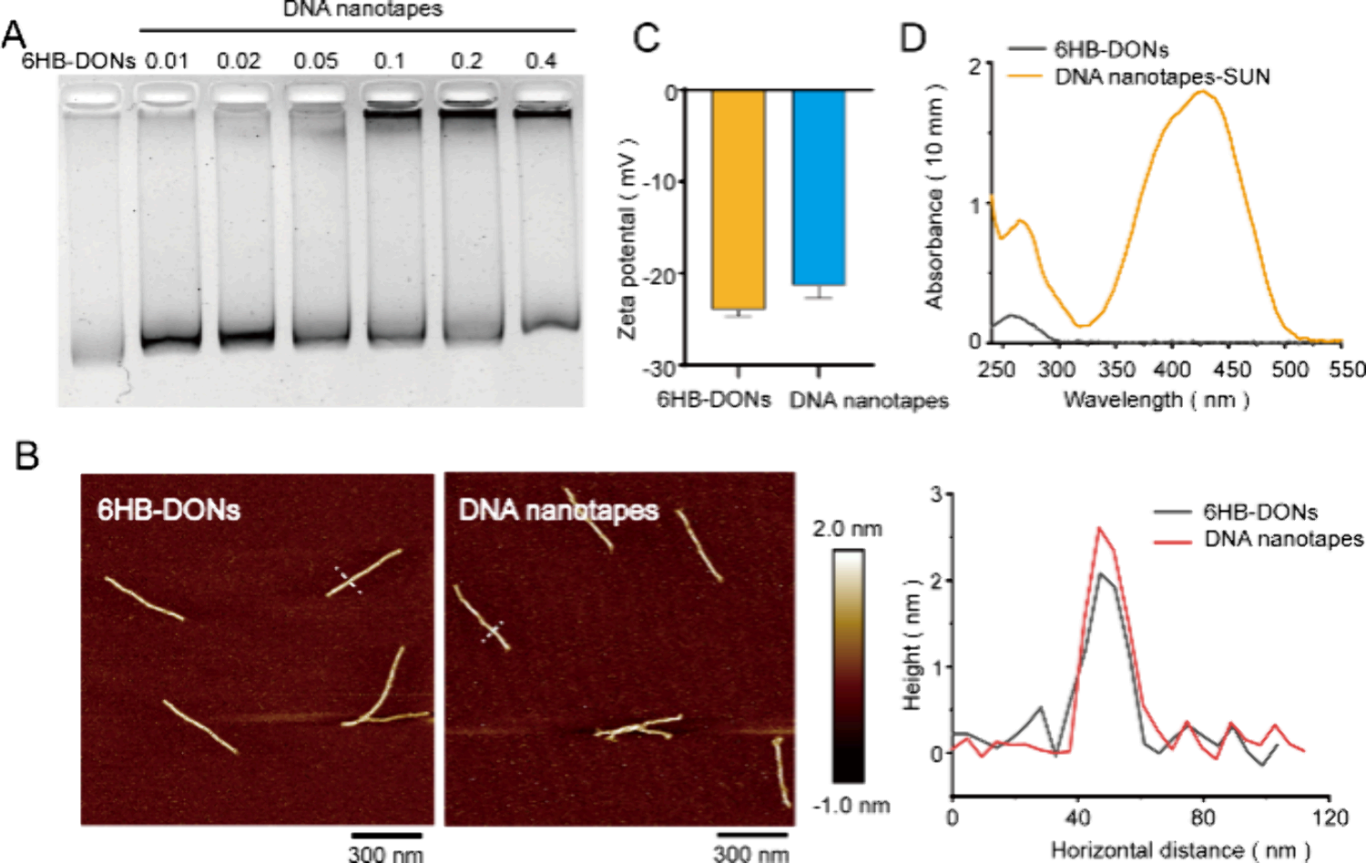
Characterization of DNA nanotape-SUN. (A) The agarose gel electrophoresis image illustrated the electrophoretic mobility shift assay for DNA nanotapes at N/P ratios of 0.01–0.4. The first lane was the reference 6HB-DONs. (B) AFM topographic images of 6HB-DONs and DNA nanotapes. Height profile along the lines crossing both 6HB-DONs (black lines) and DNA nanotapes (red lines), marked by white dashed lines on the left. Scale bars: 300 nm. (C) The ζ-potential analysis of 6HB-DONs and DNA nanotapes. (D) The UV absorption spectra of 6HB-DONs and DNA nanotape-SUN.

To achieve an optimized stoichiometry, a defined amount of DNA origami nanostructures was mixed with varying concentrations of chitosan oligosaccharide lactate (MW ≈ 5 kDa) at a specific N/P charge ratio (N/P values ranging from 0.01 to 0.4) (Table S1, Supporting Information). The gel retardation assay showed that the DNA nanotapes band moved more slowly compared to the 6HB-DONs, indicating the counterbalancing of the negative charge of phosphates upon binding to polycations and an increase in the overall size of 6HB-DONs (Figure 2A).

This could be attributed to the electrostatic interactions between the phosphate backbone of DNA origami and polycationic chitosan, resulting in the successful binding of chitosan to the surface of 6HB-DONs. When the N/P ratio increased to 0.1, two distinct bands appeared, one of which got stuck in the well of the agarose gel. It could be explained by the aggregation of 6HB-DONs resulting from the high concentration of chitosan. We performed AFM imaging of the sample with N/P equal to 0.1 and found that the 6HB-DONs were aggregated as predicted (Figure S1, Supporting Information). To avoid the aggregation of 6HB-DONs, we chose an N/P ratio of 0.05 for subsequent experiments. To validate that the nanostructure of 6HB-DONs remained unchanged in the presence of chitosan, the DNA nanotapes and 6HB-DONs were imaged by TEM and AFM. The TEM and AFM images showed that the DNA nanotapes and 6HB-DONs had the same nanostructure, with an ordered structure with a length of 380 nm (Figure 2B; Figure S2, Supporting Information). However, the height of the DNA nanotapes was higher than that of the 6HB-DONs, indicating their adsorption on the surface of 6HB-DONs. Furthermore, the ζ-potentials of 6HB-DONs and DNA nanotapes were measured to be −24.05 ± 0.67 and −21.43 ± 1.29 mV, respectively (Figure 2C). The DNA nanotapes showed a slight reduction in ζ potential compared to 6HB-DONs. During the assembly of the DNA nanotapes, the positive charge of chitosan changed the surface charge of 6HB-DONs.To validate that DNA nanotapes adsorbed SUN, DNA nanotapes were incubated with SUN and purified, and then the absorbance of the sample was measured with a UV–visible spectrophotometer (Figure 2D). The results showed that the DNA nanotape-SUN exhibited a novel absorption peak at 430 nm compared with 6HB-DONs, which was consistent with the SUN absorption peak reported in the literature,^24^ indicating that DNA nanotapes successfully adsorb SUN. The loading efficiency of SUN adsorbed on the DNA nanotapes was calculated to be 65%. Taken together, these results indicated that DNA nanotapes based on 6HB-DONs had been successfully constructed and used as a carrier for sunitinib through an electrostatic interaction.

### 3.3 SUN Delivery by DNA Nanotapes

**Figure 3:**
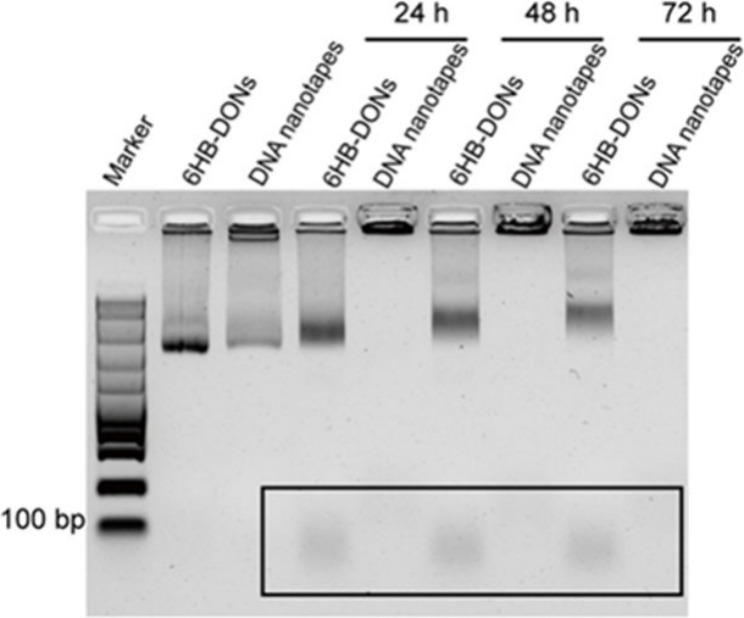
Stability analysis of DNA nanotapes. The agarose gel electrophoresis image illustrated the stability of 6HB-DONs and DNA nanotapes in the 1640 medium with 10% FBS for 24–72 h at 37 °C. The first lane was 6HB-DONs. The second lane was the DNA nanotapes. The bands within the boxes were DNA degradation bands.

Next, to evaluate the stability of DNA nanotapes under physiological conditions, DNA nanotapes and 6HB-DONs were incubated with 10% FBS in 1640 medium at 37 °C for 24 to 72 h. Agarose gel electrophoresis was used to test the serum stability of DNA nanotapes compared with that of 6HB-DONs. After FBS incubation, the agarose gel image showed that the faster bands (less than 100 bp) appeared in the 6HB-DONs group but did not appear in the DNA nanotapes group (Figure 3). Compared with untreated samples, FBS-treated DNA nanotapes were trapped in the wells of agarose gel, which may be due to the increased molecular weight of DNA nanotapes due to the adsorption of serum proteins. These results showed that chitosan shields encapsulated 6HB-DONs from nuclease degradation, indicating that DNA nanotapes were able to maintain structural stability under cell culture.

**Figure 4:**
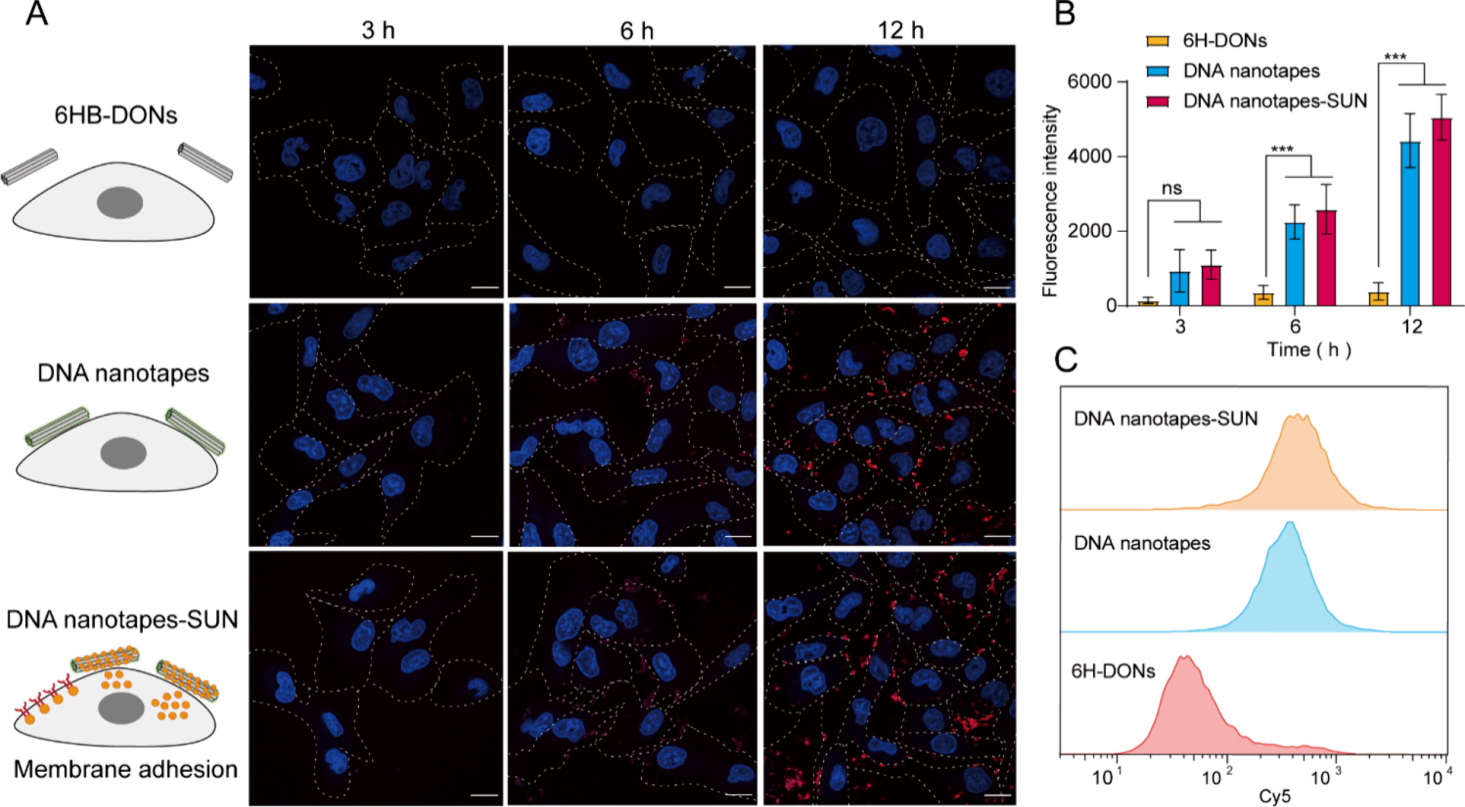
DNA nanotape-mediated SUN adsorption for renal clear carcinoma cells. (A) Confocal images of 786-0 cells incubated with 6HB-DONs, DNA nanotapes, and DNA nanotape-SUN for 3, 6, and 12 h, respectively. Scale bar, 20 μm. The cell nucleus was labeled with Hoechst 33342 (blue), and 6HB-DONs were labeled with Cy5 (red). (B) Corresponding average fluorescence intensity of 786-0 cells in confocal images. Results were compared using one-way ANOVA, *** *p* < 0.001. (C) Flow cytometry assay of the Cy5 fluorescence intensity of 786-0 cells after incubation with 6HB-DONs, DNA nanotapes, and DNA nanotape-SUN for 12 h.

To assess the cell membrane retention capacity and loading of DNA nanotape-SUN, we co-incubated Cy5-labeled DNA nanotapes with the 786-0 renal cancer cell line and traced fluorescence information at different time points. There was no obvious fluorescence signal in each group for 3 h. However, the fluorescence signal of the DNA nanotapes was enhanced compared with that of the DNA nanotube group after 6 h of incubation. Moreover, when extended to 12 h of incubation, the fluorescence signal of DNA nanotapes was further enhanced and concentrated in the cell membrane (Figure 4A,B). Notably, after long-term incubation, the Cy5 fluorescence signal was mainly concentrated on the membrane, indicating that DNA nanotapes have a cell membrane retention effect. In addition, DNA nanotapes-SUN also showed a similar fluorescence signal compared to 6HB-DONs, indicating that SUN loading did not affect the adsorption ability of DNA nanotapes to the cell membrane (Figure 4A,B). Furthermore, flow cytometry validated that DNA nanotapes and DNA nanotapes-SUN could adhere to tumor cells more efficiently than 6HB-DONs, which was consistent with the confocal imaging results (Figure 4C). Taken together, these results indicated that DNA nanotape-SUN exhibits a strong cell adhesion and cell membrane retention capacity.

### 3.4 DNA Nanotape-Mediated SUN Efficacy

**Figure 5:**
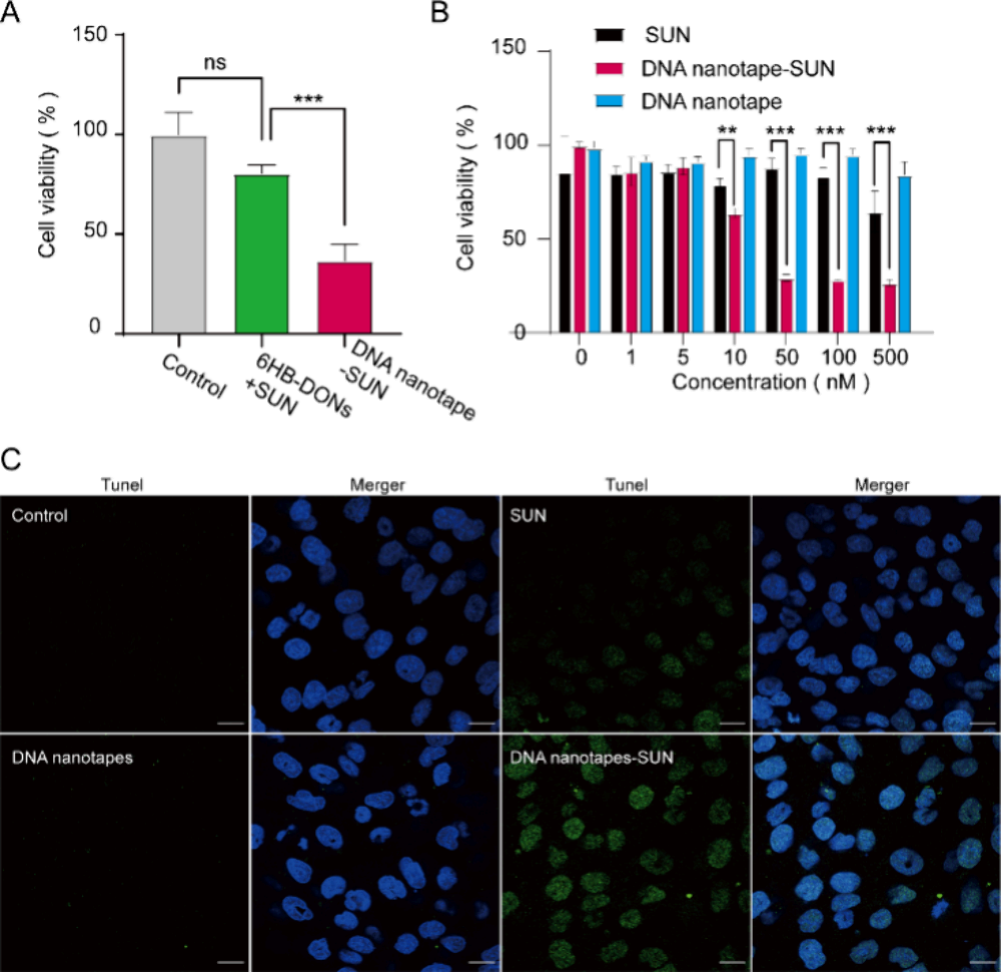
DNA nanotape-mediated SUN efficacy. (A) The cell viabilities of 786-0 cells after treatment with a mixture of 6HB-DONs, SUN, and DNA nanotape-SUN for 24 h were evaluated with a CCK8 assay. The concentration of SUN was 50 nM in both samples. Data were presented as the mean ± SD (*n* = 3). (B) The cell viabilities of 786-0 after 24 h of treatment with SUN, DNA nanotape-SUN, and DNA nanotapes were evaluated with a CCK8 assay. The concentration of SUN was between 0 and 500 nM. Data were presented as the mean ± SD (*n* = 3). Results in the left were compared using one-way ANOVA, *** *p* < 0.001. Results on the right were compared using a *t* test, **p < 0.01 and *** *p* < 0.001. (C) 786-0 cells were cultured with DNA nanotapes, SUN, and DNA nanotape-SUN for 24 h, respectively. Representative images of apoptosis in cells by the TUNEL assay. Nuclei were observed by DAPI staining, and positive staining was depicted in green. Scale bar, 20 μm.

After confirming the cell membrane adhesion and loading of DNA nanotape-SUN, we investigated their ability to kill tumor cells. 786-0 cells were incubated with DNA nanotape-SUN and 6HB-DONs mixed with SUN containing equivalent concentrations of SUN for 24 h. The CCK8 cytotoxicity assay showed that more than 75–90% of cells were viable when cultured with 6HB-DONs with SUN (Figure 5A). However, the cell viability was less than 50% in the DNA nanotape-SUN treated group compared to the group of 6HB-DONs mixed with SUN. These results indicated that the use of DNA nanotapes as a carrier for delivering SUN could increase its efficacy in renal clear carcinoma cells. Furthermore, to evaluate the enhanced therapy of DNA nanotape-SUN for tumor cells, we assessed the cell viability of 786-0 cells incubated with DNA nanotapes-SUN by the CCK8 assay, with free SUN and DNA nanotapes as the control group (Figure 5B). The CCK8 cytotoxicity assay showed that the cell viability rate in the DNA nanotapes group did not significantly decrease with increasing concentration. When treated with DNA nanotape-SUN and free SUN, the 786-0 cells exhibited different dose–response curves (Figure 5B), and the EC50 values of DNA nanotape-SUN and SUN were 10.23 and more than 500 nM, respectively. Using the TUNEL assay, we also found no TUNEL-positive cells in cells treated with DNA nanotapes, indicating that DNA nanotapes do not cause apoptosis. Furthermore, DNA nanotapes-SUN treatment significantly increased the number of TUNEL-positive cells compared with the SUN group, which was consistent with the CCK8 assay results (Figure 5C). All these results indicated that the DNA nanotape-based cell adhesion and delivery of SUN could kill renal clear carcinoma cells more efficiently.

## 4 Conclusions

We constructed DNA nanotapes with the ability to adsorb SUN on the surface, enabling long retention and RTK inhibition on the cell membrane and thereby enhancing the killing of tumor cells. These DNA nanotapes show several advantages as a drug delivery system: (1) DNA nanotapes are well-defined nanostructures. (2) DNA nanotapes serve as highly efficient carriers for anionic drugs through electrostatic adsorption and hydrogen bond interaction. (3) More importantly, DNA nanotapes can achieve cellular membrane retention and surface receptor inhibition. Taken together, this study provides a promising candidate platform for the delivery of cell membrane receptor inhibitors in anticancer therapy.

## Supporting Information Available

Pipetting scheme of DNA nanotapes at different N/P charge ratios, additional characterization of DNA nanotapes, and DNA strand sequences used in this study (Figures S1 and S2 and Tables S1–S3 (PDF)
