# Supplementary material for: DNA Origami-Constructed Nanotapes for Sunitinib Adsorption and Inhibition of Renal Clear Carcinoma Cells
Source: ACS Omega. 2024 Jul 29;9(31):33765–72. doi: 10.1021/acsomega.4c03091 (PMC11307990; doi:10.1021/acsomega.4c03091)
Supplement: Supplementary file 1 — ao4c03091_si_001.pdf [file ao4c03091_si_001.pdf]

Supporting information

DNA Origami-constructed Nanotapes for Sunitinib Adsorption and Inhibition of Renal Clear Carcinoma Cells

Lin Li[a], Xuxiang Yao[a], Pengyao Wei[a], Dongdong He[a], Qiaojiao Ding[a], Bing Bai[a], Xiuyi Lv[c], Akinori Kuzuya[d], Yuling Wang[e], Kerong Wu[c,*], Kaizhe Wang[a,*] and Jianping Zheng[a,b,*]

[a] Ningbo Key Laboratory of Biomedical Imaging Probe Materials and Technology, Ningbo Cixi Institute of Biomedical Engineering, Ningbo Institute of Materials Technology and Engineering, Chinese Academy of Sciences, Ningbo 315300, P. R. China
[b] University of Chinese Academy of Sciences, Beijing 100049, P. R. China
[c] The First Affiliated Hospital of Ningbo University, Ningbo University, Ningbo 315300, P. R. China
[d] Faculty of Chemistry, Materials, and Bioengineering, Kansai University, 3–3–35 Yamate, Suita, Osaka 564–8680, Japan
[e] School of Natural Sciences, Faculty of Science and Engineering, Macquarie University, Sydney, NSW 2109, Australia
[*]Corresponding authors

1. Preparation of DNA Nanotapes at Different N/P Charge Ratios

The formula for calculating the volume of chitosan stock solution required for preparing DNA nanotapes at different N/P charge ratios was as follows:

$$\text{Concentration of chitosan stock solution (mg/mL)} = \frac{\frac{N}{P} \times \text{nmol of all phosphates} \times \text{molecular weight of chitosan(g/mol)}}{\text{Volume (μL) of chitosan} \times \text{number of amine groups per chitosan}} \quad (1)$$

The formula for calculating the molecular weight of all phosphates was as follows:

$$\text{nmol of all phosphates} = \frac{\text{the concentration of DNA nanotube origami (ng/μL)} \times \text{μL of HB} - \text{DONs}}{\text{overage molecular of HB} - \text{DONs(g/mol)}} \quad (2)$$

the molecular weight of chitosan = 5000
number of amine groups per chitosan = 28
As an illustration, to prepare DNA nanotapes (10 μL containing 1 nmol phosphate) with chitosan at N/P 0.1, 3.6 μL of chitosan (stock concentration of 5 μg/mL) was needed:

Concentration of chitosan stock solution = $\frac{0.1\times 1\ \text{nmol}\times 5000}{3.6\times 28}$ = 5 μg/mL

Then, 6.4 μL of tris buffer was added to 3.6 μL of chitosan (5 μg/mL) to bring up the total volume to 10 μL, followed by adding 10 μL of 6HB-DONs (containing 1nmol phosphate).

Table S1. Pipetting scheme for preparing the polyplex between the 6HB-DONs (10 μL of 1 nmol phosphate) and chitosan at different N/P rations

N/P ration	Chitosan stock solution	μL of stock	μL of tris buffer

	(μg/mL)	solution to take	to take
0.01	0.5	3.6	6.4
0.02	1	3.6	6.4
0.05	2.5	3.6	6.4
0.1	5	3.6	6.4
0.2	10	3.6	6.4

2. Characterization of DNA nanotapes

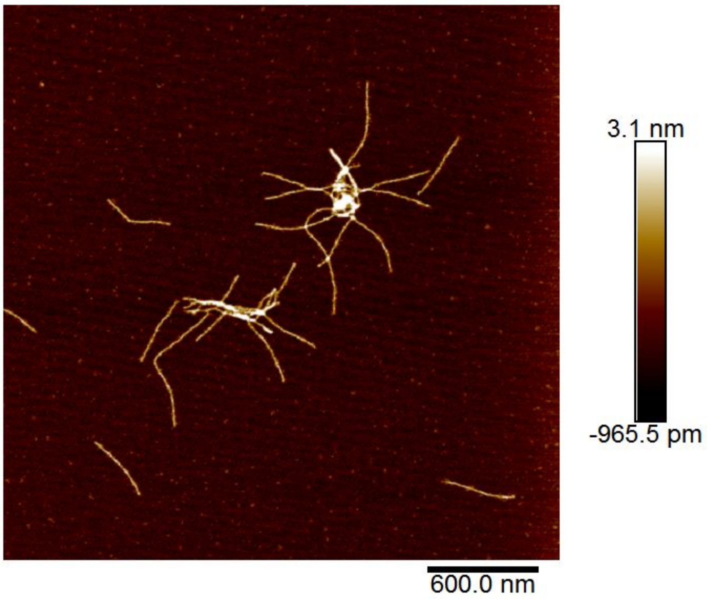

Figure S1. AFM topographic images of DNA nanotapes at N/P ratios of 0.1. Scale bars, 600 nm.

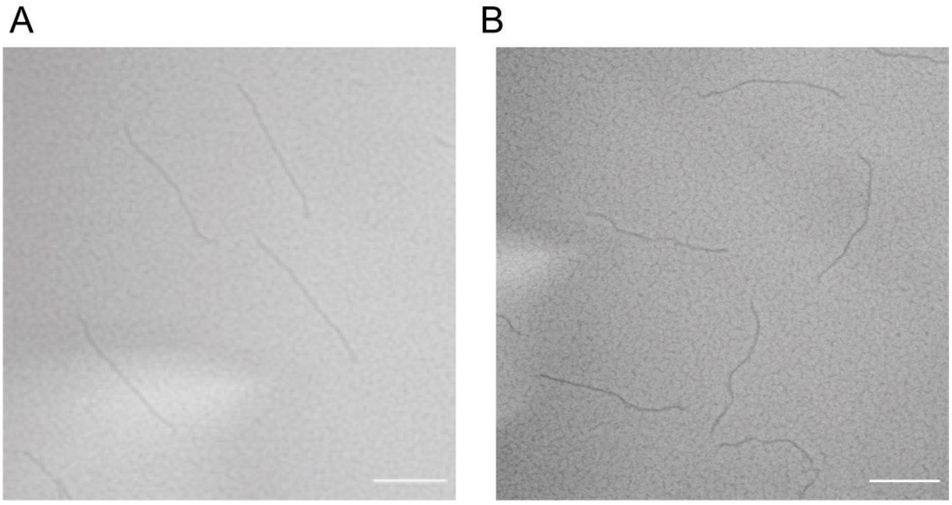

Figure S2. Representative TEM image of 6HB-DONs and DNA nanotapes. (A) TEM image of 6HB-DONs. (B) TEM image of DNA nanotapes. Scale bars, 200 nm in TEM image.

3. DNA strand sequences used in this study

Table S2. Sequences of unmodified helper strands used to assemble the 6HB-DON.

**Name**	Sequence (5'-3')

**1**	TGTGTAGGTAAAGAATAGTAGTAGCATTCCTCAGAACCGCCA
**2**	CATCAATTCTACTATTCAAAAGGGTGAGATAGATTAGAGCCG
**3**	AACCAGAGCCACCAACCCTCAGAGCCGCAGCTGAAAAGGTGG
**4**	CCCTCAGAGCCACCCCGGAACCGCCTCCAAGTATTAGACTTT
**5**	TCAATAGATAATACATTTGAGGATTTAGCTCAGAGCCGCCAC
**6**	ACAAACAATTCGACAGCACTAACAACTAAAAGGCCGGAGACA
**7**	CCTTATTAGCGTTTAGCATTGACAGGAGATGGTCAATAACCT
**8**	AAAATATCTTTAGGAACTCGTATTAAATATCAAAATCACCGG
**9**	TTATTAATTTTAAAAGTTGAAAGGAATTCCGTTCTAGCTGAT
**10**	GTCAAATCACCATCTCATTTGGGGCGCGCACCAGAACCACCA
**11**	CCAGAGCCGCCGCCGCCATCTTTTCATACCTTTGCCCGAACG
**12**	GTTTAGCTATATTTAATATGATATTCAAGAGGAAGGTTATCT
**13**	CACCGTAATCAGTAAGCGCAGTCTCTGATCTGGAAGTTTCAT
**14**	GTTGGCAAATCAACAGTTTGAGTAACATTTCGGTCATAGCCC
**15**	TGATTATCAGATGAAGCAAATGAAAAATTGGAGCAAACAAGA
**16**	AAATTAATGCCGGAGATACATTTCGCAAGTTGAGGCAGGTCA
**17**	AAGCCAGAATGGAAGCGACAGAATCAAGTATCATCATATTCC
**18**	GACGATTGGCCTTGTTTTCATCGGCATTTATCATTTTGCGGA
**19**	ACAAAGAAACCACCTATCAAACCCTCAATTGAGAGATCTACA
**20**	TGCTGAACCTCAAAAGAAGGAGCGGAATTTTGCCTTTAGCGT
**21**	AAGGCTATCAGGTCGATTCCCAATTCTGATAAATCCTCATTA
**22**	CAGACTGTAGCGCGATATTCACAAACAACGAACGAGTAGATT
**23**	TAGTTTGACCATTAGAGGGTAGCTATTTTCAATATCTGGTCA
**24**	TCCATATAACAGTTATTGCCTGAGAGTCCTAAAGCATCACCT
**25**	GGGAATTAGAGCCAGGGGTCAGTGCCTTCATTTTTGCGGATG
**26**	CAACCGATTGAGGGAGTATTAAGAGGCTAGCTTCAAAGCGAA
**27**	CGCTGAGAGCCAGCTGGCAATTCATCAAACCATCGATAGCAG
**28**	GTACCTTTTACATCACGTGGCACAGACATCAGCTCATTTTTT
**29**	GAATCGATGAACGGACTAAAGTACGGTGATTTACCGTTCCAG
**30**	TTCTGAAACATGAAAGGGAAGGTAAATAAATATACAGTAACA
**31**	TAAGCGTCATACATACGTCACCAATGAATATAATCCTGATTG
**32**	TTTGGATTATACTTAGTATTAACACCGCGCATGTCAATCATA
**33**	GAGGTGAGGCGGTCCTGAATAATGGAAGAGCACCATTACCAT
**34**	TAATAAGTTTTAACGCAAAATCACCAGTGGTTAGAACCTACC
**35**	ATATCAAAATTATTGAACGAACCACCAGCCAAAAACAGGAAG
**36**	CCATTAAAAATACCTGCACGTAAAACAGGACTTGAGCCATTT
**37**	GTATAAACAGTTAATTATCACCGTCACCAAATAAAGAAATTG
**38**	CGTAGATTTTCAGGTGCGCGAACTGATAACGTTAATATTTTG
**39**	CTATTAGTCTTTAATTTAACGTCAGATGTTGACGGAAATTAT
**40**	CTATTAGTCTTTAATTTAACGTCAGATGTTGACGGAAATTAT
**41**	TCATTAAAGGTGAATGCCCCCTGCCTATAGGATTAGAGAGTA
**42**	ATTGTATAAGCAAATTTTGATAAGAGGTGAGTAACAGTGCCC
**43**	TGTACCCCGGTTGATGCTGAATATAATGCAGGAGTGTACTGG

**44**	GCTTAGAGCTTAATTAATCAGAAAAGCCCAGAAGATAAAACA
**45**	GTTTTAAATATGCATAATCGTAAAACTACTGCAACAGTGCCA
**46**	TAGCAAGGCCGGAAGGCTTTTGATGATACTGTAGCTCAACAT
**47**	CCTTTAATTGCTCCTATTTAAATTGTAAGCCCTAAAACATCG
**48**	CCAGACCGGAAGCAAAATTTTTGTTAAAATATTTTTGAATGG
**49**	GAAAGCGTAAGAATGGGAGAAACAATAAAAAAGGGCGACATT
**50**	TAACCCACAAGAATCCAGACGTTAGTAACCAAAAGGAATTAC
**51**	AACCAATAGGAACGCGCGTTTTAATTCGGAGACTCCTCAAGA
**52**	GTTTTGTCGTCTTTTGAGTTAAGCCCAATTATCAAAATCATA
**53**	GCAACTGTTGGGAAGCAACACTATCATACGTAACGATCTAAA
**54**	AAGACTTCAAATATCCATCAAAAATAATAACCCTTCTGACCT
**55**	GGTCTGAGAGACTAATCCTGAGAAGTGTGGCCTCTTCGCTAT
**56**	GAGGCATAGTAAGAGGGCGATCGGTGCGTTTTATAATCAGTG
**57**	GAGGCATAGTAAGAGGGCGATCGGTGCGTTTTATAATCAGTG
**58**	AAGACGCTGAGAAGAAGAGTCTGTCCATGCCATTCAGGCTGC
**59**	GACGACGATAAAAAGCAAAGCGCCATTCCACGCAAATTAACC
**60**	GTTGTAGCAATACTCCTTGAAAACATAGCGAAGCCCTTTTTA
**61**	TTTTCCCTTAGAATTCTTTGATTAGTAACGGCACCGCTTCTG
**62**	AAGTTTTGCCAGAGTCCAGCCAGCTTTCTAACATCACTTGCC
**63**	TGAGTAGAAGAACTGCTTCTGTAAATCGTTACCAGAAGGAAA
**64**	GAGTGAATAACCTTCAAACTATCGGCCTGACAGTATCGGCCT
**65**	TAGCGTCCAATACTTTTGAGGGGACGACTGCTGGTAATATCC
**66**	AGAACAATATTACCTGGAAACAGTACATCCAAAAGAACTGGC
**67**	ATTACCTTTTTTAAGCCAGCCATTGCAAGGGCGCATCGTAAC
**68**	CCCCCTCAAATGCTACGTTGGTGTAGATCAGGAAAAACGCTC
**69**	ATGGAAATACCTACATTAATTACATTTAGTTAGCAAACGTAG
**70**	ATCAAGAAAACAAAATTTTGACGCTCAACGGCGGATTGACCG
**71**	ATAAATCAAAAATCTCCGTGGGAACAAATCGTCTGAAATGGA
**72**	TTATTTACATTGGCCTGAGCAAAAGAAGTAAAAGAAACGCAA
**73**	TTCATTTCAATTACAGATTCACCAGTCATGTGAGCGAGTAAC
**74**	AAGCAAAGCGGATTTCATCAACATTAAACACGACCAGTAATA
**75**	AAAGGGACATTCTGAGTTACAAAATCGCACAATCAATAGAAA
**76**	TGCTTTGAATACCAGCCAACAGAGATAGTCGCGTCTGGCCTT
**77**	CCTGTAGCCAGCTTGCATCAAAAAGATTCAGTACCAGGCGGA
**78**	AACCCGTCGGATTCAGGTCTTTACCCTGTATAGCCCGGAATA
**79**	TAATGGGATAGGTCTTAAACAGTTCAGATACCGCCACCCTCA
**80**	CGTGCATCTGCCAGGCGGAATCGTCATACAGAGCCACCACCC
**81**	CAGGAAGATCGCACGGGGTAATAGTAAAGAACCCATGTACCG
**82**	GTGCCGGAAACCAGCCAAAATAGCGAGACTACAACGCCTGTA
**83**	AACAATGAAATAGCGCCCTCATAGTTAGACCCTCGTTTACCA
**84**	GCATTCCACAGACAAATAGCTATCTTACCGATAGCTTAGATT
**85**	AGAAAAGTAAGCAGGTCACCAGTACAAAGGCTTTTGCAAAAG
**86**	TAACACTGAGTTTCATAGCCGAACAAAGTCGCTATTAATTAA

**87**	CCGAGGAAACGCAAAGCAAGCCCAATAGATGTTTAGACTGGA
**88**	TCATTTTCAGGGATTAATAACGGAATACAAATCAATATATGT
**89**	ATGATTAAGACTCCAGAACCGCCACCCTAATATTCATTGAAT
**90**	GAACCGCCACCCTCTTATTACGCAGTATACAATTTCATTTGA
**91**	AAAATACATACATACTCAGGAGGTTTAGAAACGAGAATGACC
**92**	GGTGTATCACCGTAAAGGTGGCAACATAATGATGAAACAAAC
**93**	AGACACCACGGAATAGGGTTGATATAAGACTATTATAGTCAG
**94**	TAAGTGCCGTCGAGAAGTTTATTTTGTCGCAGAGGCGAATTA
**95**	ATTCATATGGTTTAAGCGGGGTTTTGCTAAGAGGAAGCCCGA
**96**	GAAGGATTAGGATTCCAGCGCCAAAGACCGGATTCGCCTGAT
**97**	AACGGTACGCCAGACCTTTTTAACCTCCTAATATCAGAGAGA
**98**	GAACGCGAGGCGTTGCATCGGAACGAGGAGGCTGGCTGACCT
**99**	TACGCCAGCTGGCGGCAGATACATAACGATGAATTTTCTGTA
**100**	AGCAGCGAAAGACATTAGCGAACCTCCCCCAACATGTAATTT
**101**	CTGCCCGCTTTCCACTTGACAAGAACCGGGATCGTCACCCTC
**102**	CACATTCAACTAATAAAGGGGGATGTGCGGATTTTAGACAGG
**103**	AGGCAGAGGCATTTCCATCACCCAAATCCGTGCCAGCTGCAT
**104**	TCATCAAGAGTAATGTCGGGAAACCTGTAAGTTTTTTGGGGT
**105**	CGAGGTGCCGTAAACATATTTAACAACGGACTTGCGGGAGGT
**106**	TAATTGAGAATCGCGCACTAAATCGGAATTGCGTTGCGCTCA
**107**	AAATCAACGTAACACTAACTCACATTAACCCTAAAGGGAGCC
**108**	CCCGATTTAGAGCTTATAAAGCCAACGCTATTTTGCACCCAG
**109**	CAAATTCTTACCAGTGACGGGGAAAGCCGTAAAGCCTGGGGT
**110**	CCCTGACGAGAAACCGGAAGCATAAAGTGGCGAACGTGGCGA
**111**	GAAAGGAAGGGAAGAAGCCTGTTTAGTAGCTAACGAGCGTCT
**112**	ATAATTACTAGAAAAAAGCGAAAGGAGCCGCTCACAATTCCA
**113**	GATGGTTTAATTTCGTGAAATTGTTATCGGGCGCTAGGGCGC
**114**	TGGCAAGTGTAGCGGCGTTAAATAAGAAATAAACAGCCATAT
**115**	GTGTGATAAATAAGGTCACGCTGCGCGTCGTAATCATGGTCA
**116**	CGATTTTAAGAACTACCGAGCTCGAATTAACCACCACACCCG
**117**	CCGCGCTTAATGCGCTAAATTTAATGGTTTTTTTGTTTAACG
**118**	TTTCATCTTCTGACCCGCTACAGGGCGCGCAGGTCGACTCTA
**119**	AAGAAAAATCTACGAAGCTTGCATGCCTGTACTATGGTTGCT
**120**	TTGACGAGCACGTAAAAACTTTTTCAAAAGAGAATAACATAA
**121**	ACAAAGAACGCGAGTAACGTGCTTTCCTACGACGTTGTAAAA
**122**	TATTACAGGTAGAAGGGTTTTCCCAGTCCGTTAGAATCAGAG
**123**	CGGGAGCTAAACAGTAAATGCTGATGCATTAACTGAACACCC
**124**	TATATAACTATATGGAGGCCGATTAAAGTGCAAGGCGATTAA
**125**	GTTGGGTAACGCCAAGATTCATCAGTTGTTTCAGCGGAGTGA
**126**	CGACGGCCAGTGCCTTAATAAAACGAACCGAATAATAATTTT
**127**	GAGGATCCCCGGGTGGCTCATTATACCACTCCAAAAGGAGCC
**128**	TAGCTGTTTCCTGTAACTTTAATCATTGTTCGAGGTGAATTT
**129**	CACAACATACGAGCACCAGAACGAGTAGCCGACAATGACAAC

**130**	GCCTAATGAGTGAGAAGCTGCTCATTCATCGGTCGCTGAGGC
**131**	TTTGAAGCCTTAAAAGGCCGCTTTTGCGGATATTCATTACCC
**132**	TTGCAGGGAGTTAATCAAGATTAGTTGCTCAACAGTAGGGCT
**133**	CTACAATTTTATCCCATAACCGATATATGTGAATAAGGCTTG
**134**	AACCATCGCCCACGTGAATCTTACCAACTCATATGCGTTATA
**135**	TTCCAGAGCCTAATTACCGATAGTTGCGTAAATTGGGCTTGA
**136**	CTTAAACAGCTTGATTGCCAGTTACAAATAAACACCGGAATC
**137**	TATTTATCCCAATCTTTATCAGCTTGCTTGAATTACCTTATG
**138**	TTTAATTGTATCGGCAAATAAGAAACGATTGAAATACCGACC
**139**	TCAAAAATGAAAATCTCCAAAAAAAAGGGTCAGGACGTTGGG
**140**	TTCACGTTGAAAATAGCAGCCTTTACAGTATATTTTAGTTAA
**141**	AAACAGGGAAGCGCAACTAAAGGAATTGTAACGGAACAACAT
**142**	GAATAGAAAGGAACATTAGACGGGAGAAAATCCAATCGCAAG
**143**	TGAACAAAGTCAGAACAACTTTCAACAGAGATTTAGGAATAC
**144**	TGGGATTTTGCTAAGGGTAATTGAGCGCGGCTTAGGTTGGGT
**145**	GCCCACTACGTGAATCGAGCCAGTAATAATCCGGTATTCTAA
**146**	TAATGAATCGGCCACAGACCAGGCGCATGTAGCAACGGCTAC
**147**	AGATGAACGGTGTAACGCGCGGGGAGAGCTATCAGGGCGATG
**148**	TTTACGAGCATGTATTTGATGGTGGTTCAGGCGAAAATCCTG
**149**	CGGAGATTTGTATCTGGTTTGCCCCAGCCGAAATCGGCAAAA
**150**	TCCCTTATAAATCAGAACAAGAAAAATACTTATCATTCCAAG
**151**	AATAGATAAGTCCTAAAGAATAGCCCGATGAGAGAGTTGCAG
**152**	CGACCTGCTCCATGTCACCGCCTGGCCCGATAGGGTTGAGTG
**153**	TTGTTCCAGTTTGGCTAATGCAGAACGCATCGAGAACAAGCA
**154**	AACAACATGTTCAGAACAAGAGTCCACTAGTGAGACGGGCAA
**155**	TCAATCATAAGGGATTTTTCTTTTCACCATTAAAGAACGTGG
**156**	ACTCCAACGTCAAAAAGTAATTCTGTCCATTACCGCGCCCAA
**157**	ACCGACAAAAGGTAGGGCGAAAAACCGTGCGGTTTGCGTATT
**158**	GGGCGCCAGGGTGGACCGAACTGACCAAATGAGGAAGTTTCC
**159**	CAGCTGATTGCCCTTTACTTAGCCGGAATACGAAGGCACCAA
**160**	CAAGCGGTCCACGCATCGCCTGATAAATCTAAAACACTCATC
**161**	GAAACCAATCAATAATTATACCAAGCGCGAAACAAAGTACAA
**162**	TTTGACCCCCAGCGATCGGCTGTCTTTCATATCCCATCCTAA
**163**	AACGGGTATTAAACGGCAAAAGAATACATGTGTCGAAATCCG
**164**	CCTAAAACGAAAGACAAGTACCGCACTCGCCTGTTTATCAAC
**165**	AGCCGTTTTTATTTATACGTAATGCCACCGAGGCGCAGACGG
**166**	ATTAAACGGGTAAATCATCGTAGGAATCAGACGACGACAATA
**167**	TAGCAAGCAAATCACTAAAGACTTTTTCCTTTGAAAGAGGAC
**168**	AGAGGCTTTGAGGAGATATAGAAGGCTTAGAGAATATAAAGT

Table S3. Sequences of helper strands functionalized with the single-stranded DNA capture probe. Strand names refer to unmodified helper strands listed in Table S2. The 17 bases region

complementary to the Cy5-DNA conjugate is highlighted in red.

Name	Sequence (5'-3')
**4**	CCCTCAGAGCCACCCCGGAACCGCCTCCAAGTATTAGACTTT<br>CGTTGTTGAGTCAACGGCCT
**8**	AAAATATCTTTAGGAACTCGTATTAAATATCAAAATCACCGG<br>CGTTGTTGAGTCAACGGCCT
**12**	GTTTAGCTATATTTAATATGATATTCAAGAGGAAGGTTATCT<br>CGTTGTTGAGTCAACGGCCT
**16**	AAATTAATGCCGGAGATACATTTCGCAAGTTGAGGCAGGTCA<br>CGTTGTTGAGTCAACGGCCT
**20**	TGCTGAACCTCAAAAGAAGGAGCGGAATTTTGCCTTTAGCGT<br>CGTTGTTGAGTCAACGGCCT
**24**	TCCATATAACAGTTATTGCCTGAGAGTCCTAAAGCATCACCT<br>CGTTGTTGAGTCAACGGCCT
**28**	GTACCTTTTACATCACGTGGCACAGACATCAGCTCATTTTTT<br>CGTTGTTGAGTCAACGGCCT
**32**	TTTGGATTATACTTAGTATTAACACCGCGCATGTCAATCATA<br>CGTTGTTGAGTCAACGGCCT

Cy5 single strand：AGGCCGTTGACTCAACG-Cy5
